# BRG1 knockdown inhibits proliferation through multiple cellular pathways in prostate cancer

**DOI:** 10.1186/s13148-021-01023-7

**Published:** 2021-02-17

**Authors:** Katherine A. Giles, Cathryn M. Gould, Joanna Achinger-Kawecka, Scott G. Page, Georgia R. Kafer, Samuel Rogers, Phuc-Loi Luu, Anthony J. Cesare, Susan J. Clark, Phillippa C. Taberlay

**Affiliations:** 1 Epigenetics Laboratory, Genomics and Epigenetics Theme, Garvan Institute of Medical Research, Sydney, NSW 2010 Australia; 2 Genome Integrity Unit, Children’s Medical Research Institute, University of Sydney, Westmead, NSW 2145 Australia; 3 Tasmanian School of Medicine, College of Health and Medicine, University of Tasmania, TAS, Hobart, 7000 Australia; 4 St Vincent’s Clinical School, UNSW Sydney, Sydney, NSW 2000 Australia

**Keywords:** BRG1, *SMARCA4*, Chromatin remodelling, Cancer, Gene expression, Cell cycle, Transcription, DNA replication

## Abstract

**Background:**

BRG1 (encoded by *SMARCA4*) is a catalytic component of the SWI/SNF chromatin remodelling complex, with key roles in modulating DNA accessibility. Dysregulation of BRG1 is observed, but functionally uncharacterised, in a wide range of malignancies. We have probed the functions of BRG1 on a background of prostate cancer to investigate how BRG1 controls gene expression programmes and cancer cell behaviour.

**Results:**

Our investigation of *SMARCA4* revealed that BRG1 is over-expressed in the majority of the 486 tumours from The Cancer Genome Atlas prostate cohort, as well as in a complementary panel of 21 prostate cell lines. Next, we utilised a temporal model of BRG1 depletion to investigate the molecular effects on global transcription programmes. Depleting BRG1 had no impact on alternative splicing and conferred only modest effect on global expression. However, of the transcriptional changes that occurred, most manifested as down-regulated expression. Deeper examination found the common thread linking down-regulated genes was involvement in proliferation, including several known to increase prostate cancer proliferation (*KLK2*, *PCAT1* and *VAV3*). Interestingly, the promoters of genes driving proliferation were bound by BRG1 as well as the transcription factors, AR and FOXA1. We also noted that BRG1 depletion repressed genes involved in cell cycle progression and DNA replication, but intriguingly, these pathways operated independently of AR and FOXA1. In agreement with transcriptional changes, depleting BRG1 conferred G1 arrest.

**Conclusions:**

Our data have revealed that BRG1 promotes cell cycle progression and DNA replication, consistent with the increased cell proliferation associated with oncogenesis.

**Supplementary Information:**

The online version contains supplementary material available at 10.1186/s13148-021-01023-7.

## Background

Nucleosomes serve as a physical backbone for chromatin organisation on a global scale and at local gene regulatory elements. Nucleosomes therefore govern both genome-wide stability and local DNA accessibility [1]. Nucleosome positioning by ATP-dependent chromatin remodellers plays a critical role in regulating DNA accessibility and allows genes to be expressed at the appropriate place and time [1]. Genomic profiling has demonstrated that dynamic regulation of DNA accessibility occurs primarily at DNA regulatory elements, which are cell type specific, and that DNA accessibility changes reflect concomitant transcriptional patterns [2, 3]. It is essential for chromatin to be relaxed at active gene promoters to create an ordered nucleosome disassembly, which permits binding of RNA pol II and the general transcription machinery [4, 5]. In agreement, ChIP-seq data show that transcription factors are concentrated on accessible DNA, with the highest levels of bound transcription factors correlating with the most accessible genomic regions [6]. Conversely, chromatin condensation resulting in reduced DNA accessibility is necessary for transcriptional repression [7]. Disruption to the DNA accessibility landscape is a feature of cancer [2, 8, 9]. This was recently emphasised in genomic sequencing data from multiple cancers and cancer subtypes, which revealed associations between the accessible chromatin organisation and mutation load [8]. Moreover, studies of aged human and yeast cells demonstrated that nucleosome loss compromises genome stability, gene regulation and transcription [10, 11].

Genes encoding ATP-dependent chromatin remodellers are themselves frequently mutated and often atypically expressed in cancer [5, 12–16]. Notably, the SWI/SNF (switch/sucrose non-fermentable) chromatin remodelling complex is mutated or transcriptionally deregulated in ~ 20% of cancers; a mutation frequency approaching that of *TP53* (~ 26%) [12, 14, 17]. The SWI/SNF complex is often described as a tumour suppressor because it is required by the Retinoblastoma protein (Rb) family for regulation of normal cell growth [18, 19]. Disruptions of multiple SWI/SNF subunits are reported in human tumours and cell lines [13–15, 20–37], often accompanied by a loss of heterozygosity consistent with the inactivation of a tumour suppressor [13, 34]. The specific SWI/SNF mutations observed in tumours and the cancers associated with altered SWI/SNF function have been extensively reviewed [12–15, 26, 31, 34, 38]. However, the mechanism and functional consequences of SWI/SNF dysregulation are still being defined.

Brahma-related gene 1 (BRG1) is one of the two mutually exclusive ATPases within the SWI/SNF complex. Interestingly, *SMARCA4 *(*SWI/SNF-related, matrix-associated, actin-dependent regulator of chromatin, Subfamily A, Member 4*), the gene encoding BRG1, has been observed in both down- and up-regulated states in cancer, indicative of the diverse and complex BRG1 functions. *SMARCA4* mRNA was seen to be down-regulated in bladder, colon, non-triple negative breast cancers, head and neck, oesophageal, melanoma, pancreatic, lung and ovarian cancers, and *SMARCA4* mutation rates in these cancers have been reported between 4 and 13% [12–14, 22, 24, 30, 39–41]. In contrast, *SMARCA4* has been reported as over expressed in cancers of the prostate, triple negative breast cancers and some leukaemias [12, 22, 24, 30, 42, 43]. In *SMARCA4* over expressing cancers, no significant recurrent mutations have been reported [42, 44–46]. The importance of BRG1 in cancer is further evidenced through studies of synthetic lethality, where BRG1 was observed to have a synthetic lethal relationship with the alternative SWI/SNF ATPase Braham (BRM), and Aurora A kinase in lung cancer, and PTEN in prostate cancer [43, 47, 48].

Examination of multiple prostate cancer cohorts has demonstrated elevated *SMARCA4* expression or increased BRG1 protein levels. Clinical studies of primary prostate tumours reported an overall increase in BRG1 protein by immunohistochemistry [42–46], and increased *SMARCA4* gene expression has been reported in tumours from The Cancer Genome Atlas (TCGA) prostate cancer cohort compared to normal prostate tissue [49, 50]. Moreover, higher *SMARCA4* gene expression or increased BRG1 protein levels are inversely correlated with patient survival [43, 49]. While it is established that BRG1 is commonly up-regulated in prostate cancer, the full range of molecular pathways impacted by dysregulated BRG1 levels and the contribution of these molecular changes to the atypical phenotype of prostate cancer cells remains unclear.

BRG1 has known roles in regulating DNA for temporal gene expression at both promoters and enhancer gene regulatory elements [4, 51–56]. Moreover, BRG1 maintains the epigenetic landscape of a cell at these gene regulatory elements. Specifically, BRG1 has been directly linked to transcriptional output through its recognition of H3K14ac [57–59]. In the absence of H3K14ac, BRG1 is still present at promoters and histones are disassembled from the chromatin; however, transcription is reduced [60]. At enhancers, BRG1 depletion greatly reduces H3K27ac and subtlety reduces H3K4me1, which is correlated with a decrease in chromatin accessibility [53]. BRG1 is also known to mediate inter-chromosomal looping interactions between specific loci such as the *MYC* enhancer and promoter, the alpha-globulin genes, the *IgH* locus and the class II major histocompatibility complex gene locus [24, 61–64]. On a global scale, BRG1 binding has been found at DNA-loop anchors [56] and topological associated domain (TAD) boundaries where it increases their stability [65]. Together, this demonstrates an important role for BRG1 in maintaining chromatin architecture at both local and global levels for transcription regulation.

Here, we dissected the molecular role of BRG1 on the transcriptome in prostate cancer. We confirmed that *SMARCA4* is over-expressed in prostate cancer irrespective of molecular subtype, and identified *SMARCA4* was also over expressed in a panel of prostate cancer cell lines. Depletion of BRG1 in LNCaP prostate cancer cells resulted in a modest effect on global gene transcription with most changes resulting in down-regulated gene expression. Within the cohort of down-regulated genes in BRG1 depleted cells, we identified gene clusters defined by their co-occupancy or independence from the androgen receptor (AR) and Forkhead box A1 (FOXA1) transcription factors, both of which are known BRG1 co-activators [66–68]. Our data revealed that BRG1, AR and FOXA1 co-regulate known prostate cancer genes *KLK2*, *PCAT1* and *VAV3*. Gene ontology analysis further revealed that genes regulated by BRG1 independent of AR and FOXA1 include factors regulating cell cycle and proliferation processes including DNA replication. In agreement, depleting BRG1 promoted G1 arrest resulting in reduced cell proliferation. Cumulatively, the data indicate that BRG1 promotes expression of cellular proliferation factors and cancer-associated genes in prostate cancer cells.

## Results

### *SMARCA4* is over expressed in prostate cancer irrespective of molecular subtype

**Fig. 1:**
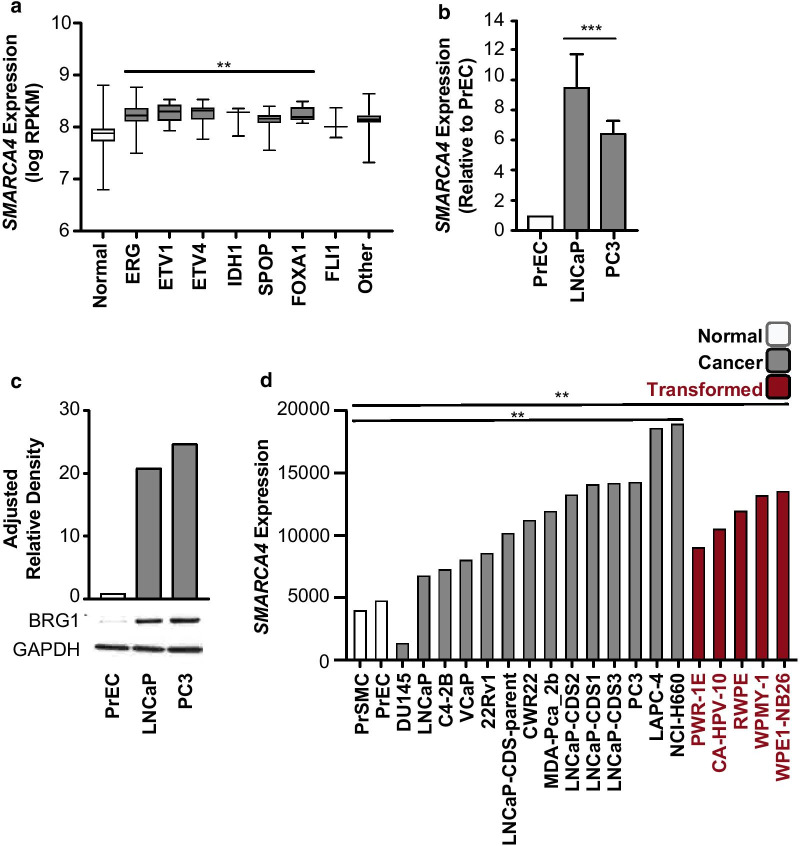
*SMARCA4 *(BRG1) is over expressed in prostate cancer. **a** *SMARCA4* gene expression (logPRKM) in TCGA data (tumours n = 486, normal = 52) with tumour samples separated by molecular subtype defined by the TCGA. *SMARCA4* expression is increased across all groups, with subtypes ERG, ETV1, ETV4, IDH1, SPOP and FOXA1 all significantly up-regulated, one-way ANOVA Dunnett’s multiple comparison correction **p < 0.05. **b** *SMARCA4* gene expression in prostate cell lines normalised to *18S* and relative to PrEC (n = 2). Significance determined by one-way ANOVA with Tukey’s multiple comparison correction ***p < 0.001. Bars denote mean, and error bars are SD. **c** Representative Western blot of BRG1 protein level in prostate cell lines. Quantification above Western blot by adjusted relative density normalised to GAPDH and relative to PrEC. **d** Expression of *SMARCA4* from RNA-seq in prostate cell lines grouped as normal, cancer or transformed. The mean of each group was calculated, and a significance was tested by one-way ANOVA Dunnett’s multiple comparison correction, **p < 0.05

We first examined the expression of *SMARCA4* in the TCGA [50] prostate normal and cancer cohort. The 486 tumour samples were subset into the seven TCGA categorised molecular subtypes of prostate cancer [50]. These included those with fusion genes involving *ERG* (46%), *ETV1* (8%), *ETV4* (4%) and *FLI1* (1%), or those with mutations in *SPOP* (11%), *FOXA1* (3%) or *IDH1* (1%) [50]. The remaining samples were grouped as ‘other’ (26%). Each subtype exhibited a statistically significant increase in *SMARCA4* expression (*p* < 0.05) with the exception of the ‘FLI1′ subtype (*p* = 0.5899) and ‘other’ (*p* = 0.1899), which both demonstrated a non-significant increase in *SMARCA4* expression (Fig. 1a). Previous work examining *SMARCA4* expression in the TCGA prostate cancer cohort demonstrated that it is also up-regulated irrespective of Gleason score [49]. Therefore, we conclude that at the mRNA level, *SMARCA4* is universally over-expressed in prostate cancer, regardless of clinical grade or molecular subtype.

### *SMARCA4* is over expressed in prostate cancer and transformed prostate cell lines

We next examined both BRG1 protein and *SMARCA4* gene expression levels in normal prostate epithelial cells (PrEC) and compared to LNCaP (lymph node metastasis), an androgen-sensitive prostate cancer cell line, as well as PC3 (bone metastasis), an androgen-insensitive prostate cancer cell line. We found that *SMARCA4* gene expression was increased ~ ninefold in LNCaP cells and ~ sixfold in PC3 compared to PrEC (*p* < 0.001; Fig. 1b). Further, the BRG1 protein level was increased ~ 20 and ~ 24 fold, respectively, in each of the prostate cancer cell lines compared to PrEC (Fig. 1c). We compared this to published RNA-seq data of several normal, cancer and transformed prostate cell lines [69]. The mean expression of *SMARCA4* was significantly increased in both the cancer cell lines and the transformed cell lines compared to the normal cells (*p* = 0.0148 and *p* = 0.0353, respectively; Fig. 1d). The exception was DU145 cells that has a known frameshift mutation in *SMARCA4,* resulting in reduced expression [36]. These data show that common prostate cancer cell lines reflect the same pattern of increased BRG1 protein that is observed in prostate tumours compared to normal prostate samples and therefore provides an appropriate model system to explore the functional consequences of BRG1 dysregulation on the transcriptome.

### BRG1 is required for the maintenance of active gene expression

**Fig. 2:**
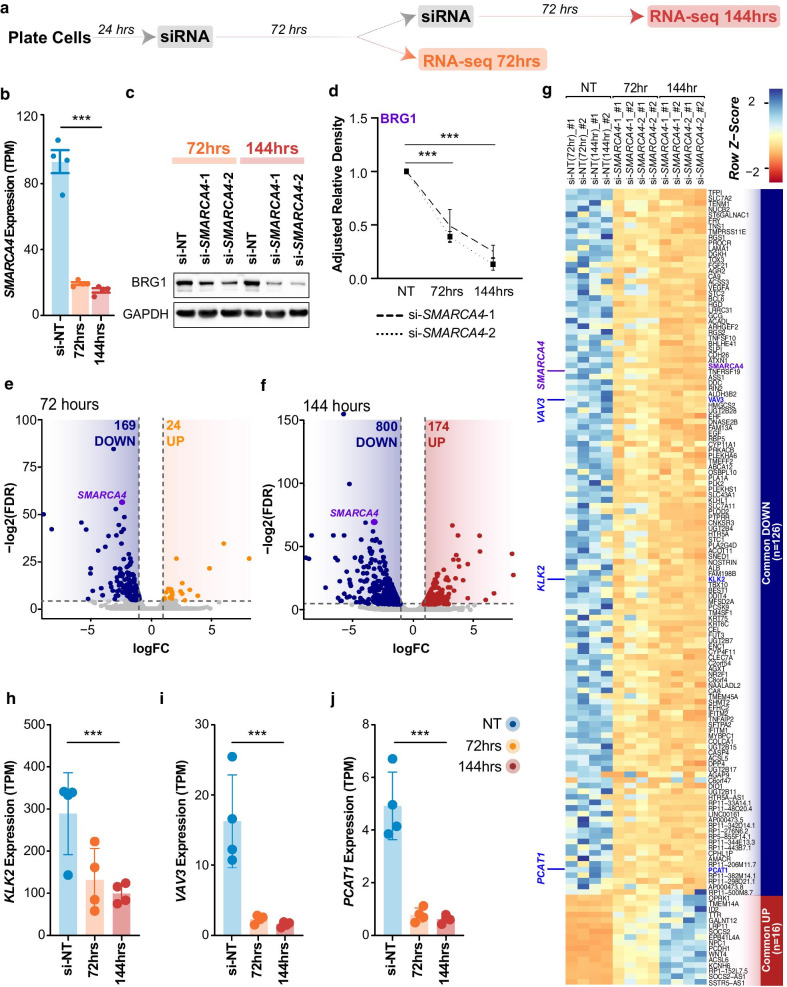
Loss of BRG1 results in a down-regulation of gene expression. **a** Schematic of temporal BRG1 knockdown model used for RNA-seq. Samples were collected at 72hrs (si-NT control, si-*SMARCA4*-1 and si-*SMARCA4*-2) and 144hrs (si-NT, si-*SMARCA4*-1 and si-*SMARCA4*-2) post-siRNA transfection in duplicate for each condition at each time point (n = 2). Cells were transfected with either control siRNA (si-NT) or *SMARCA4* siRNA. **b** *SMARCA4* gene expression in control and post BRG1 depletion in the RNA-seq data, shown as transcripts per million reads (TPM). Control siRNA for 72 and 144 h is shown collectively as si-NT. *SMARCA4* expression is significantly down-regulated at both time points, ***p < 0.0001. Bars denote mean, and error bars are SD. **c** Representative Western blots of BRG1 and GAPDH protein levels at 72 and 144 h post-transfection. **d** Adjusted relative density for BRG1 is calculated relative to GAPDH and normalised to the non-targeting control. Points denote mean, and error bars are SD. **e**, **f** Volcano plots of differentially expressed genes at 72 h and 144 h post-knockdown. Significantly down-regulated genes are blue and significantly up-regulated genes for 72 and 144 h post-knockdown are shown in orange and red, respectively. *SMARCA4* differential expression is highlighted in purple. Expression is shown as normalised log2 counts per million reads. **g** Heatmap illustrating RNA-seq differential gene expression data for up (n = 16) and down (n = 126) regulated genes common to both time points after BRG1 depletion. Expression is represented as the normalised row Z-score of TPM. **h, i, j** *KLK2, VAV3 and PCAT-1* gene expression from the RNA-seq datasets shown as TPM. Bars denote mean, and error bars are SD

Our previous work has shown that BRG1 occupancy is enriched at active promoter and enhancer gene regulatory elements in LNCaP cells [56]. We therefore hypothesised that BRG1 would play an important role in maintaining the transcriptional profile of these cells. To assess this, we depleted the level of BRG1 protein using two independent siRNAs targeting *SMARCA4* (si-*SMARCA4*-1 and si-*SMARCA4*-2) and performed RNA-seq at 72 and 144 h post-transfection (Fig. 2a). Our RNA-seq data confirmed successful depletion of the *SMARCA4* transcript (~ 80%) at both time points (Fig. 2b). To confirm a decrease in BRG1 function, we performed a Western blot for BRG1 protein levels. We found BRG1 protein levels reduced to ~ 50% of the non-targeting control at 72 h, and to ~ 20% of the non-targeting control at 144 h post-transfection (Fig. 2c, d). We note that there were no significant changes detected in the gene expression of any other SWI/SNF subunit proteins (Additional file 1: Figure 1A). Further quality assessment of the RNA-seq data through a principal component analysis demonstrated that the samples separated by time-point on the first dimension, accounting for 43.39% of the sample variance (Additional file 1: Figure 1B). We performed a differential gene expression analysis and identified 169 down-regulated genes and 24 up-regulated genes (logFC > 1.5, FDR < 0.05) at 72 h post BRG1 depletion (Fig. 2e). This increased to 800 down-regulated genes and 174 up-regulated genes by 144 h post-transfection (Fig. 2f). This suggests that the primary role of BRG1 in LNCaP cells is to maintain active gene expression of a subset of genes.

### BRG1 does not function in the regulation of alternative splicing

The nucleosome barrier within genes is reported to contribute to alternative splicing, where there is a higher conservation of nucleosomes at the splice sites of constitutive exons compared to skipped exons [70–72]. Since the contribution of BRG1 to alternative splicing regulation is unknown, we investigated whether this may contribute to the BRG1-dependent changes in gene expression. To do this, we performed a multivariate analysis of transcript splicing (MATS; [73–75]) of our entire RNA-seq datasets. After 72 h of BRG1 depletion, MATS pairwise comparison detected a genome wide total of 13 and 11 skipped exons, and 14 and 9 retained introns with si-*SMARCA4*-1 and si-*SMARCA4*-2, respectively (Additional file 1: Figure 1C). At 144 h post BRG1 knockdown, this increased to 240 and 260 skipped exons, and 27 and 26 retained introns with si-*SMARCA4*-1 and si-*SMARCA4*-2, respectively (Additional file 1: Figure 1D). Given the relatively large number of intron–exon junctions within the total LNCaP transcriptome, we conclude that BRG1 does not extensively contribute to alternative splicing as the mechanism for alterations in gene expression. However, we do note that at 144 h post-knockdown, the MATS analysis identified retention of the first intron from the Kallikrein 3 gene, which encodes prostate specific antigen (PSA) (Additional file 1: Figure 1E). This splice variant has previously been reported in LNCaP cells and generates a unique protein from canonical PSA [76]. While PSA has a well-known link to prostate cancer, the function of its alternative splice variant remains unknown.

### BRG1 binding is associated with expression of prostate cancer associated genes

We further examined our RNA-seq datasets to determine which genes showed a significant change in expression at 72 h that was maintained at 144 h. Of the genes that were down-regulated at the 72 h time point, 126 genes (75%) remained down-regulated at 144 h. Similarly, of the up-regulated genes, 16 (67%) remained up-regulated at the extended time point (Fig. 2g). Within the down-regulated gene set, we note a number of genes that have previously been associated with increased proliferation in prostate cancer; these include kallikrein 2 (*KLK2*), long non-coding RNA prostate cancer associated transcript 1 (*PCAT1*), Vav guanine nucleotide exchange factor 3 (*VAV3*) [69, 77–84] (Fig. 2h–j). We also examined the panel of prostate cell lines [69] and confirmed that, on average there is elevated expression of these genes in both prostate cancer cells and transformed prostate cell lines compared to normal prostate cells (Additional file 2: Figure 2A). This suggests a role for BRG1 in maintaining the expression of genes associated with prostate cancer proliferation.

**Fig. 3:**
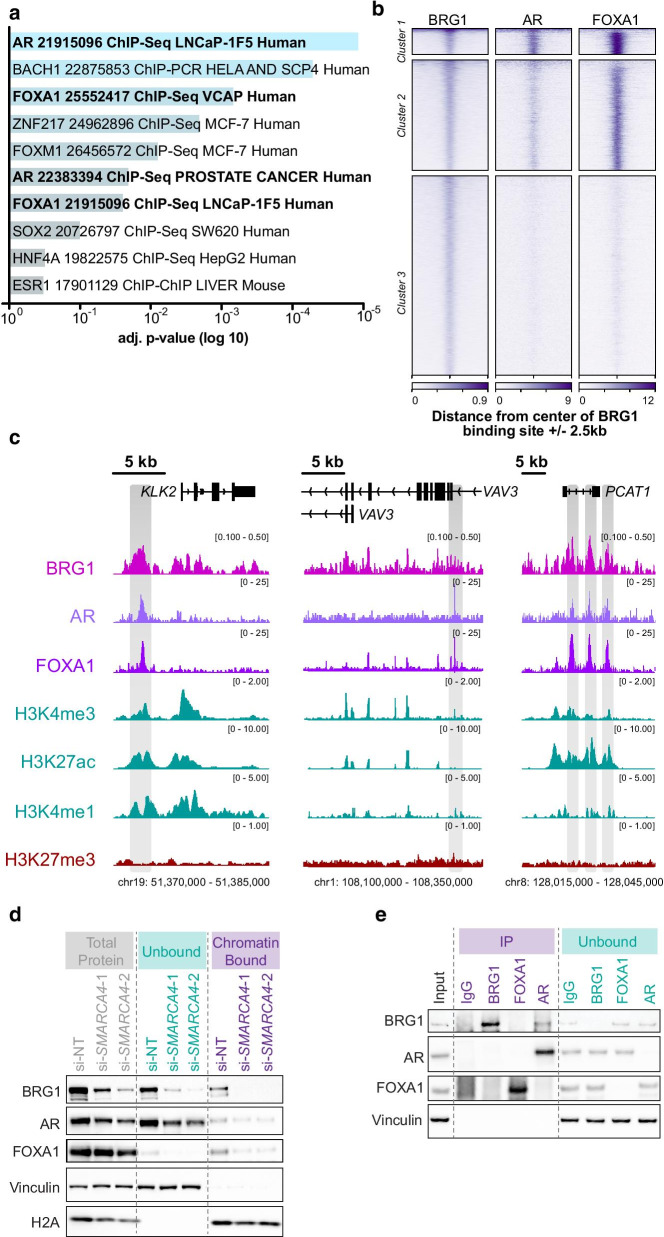
BRG1 regulates genes associated with prostate cancer. **a** Gene set enrichment analysis using ‘Enrichr’ of differentially expressed genes that are common to both time points, showing the adjusted *p* value (log 10, reversed x-axis) of significantly enriched transcription factor ChIP-seq from ChEA curated data (*p* < 0.05). **b** Heatmap of BRG1, AR and FOXA1 ChIP-seq signal at BRG1 binding sites genome-wide in LNCaP cells, ± 2.5 kb from the centre of the binding site. Data are clustered into three groups by k-means. **c** IGV images of the genes *KLK2*, *PCAT-1* and *VAV3*. Grey shaded regions contain ChIP-seq signal peaks for BRG1, AR and FOXA1. **d** Representative Western blot of the abundance of soluble unbound proteins versus chromatin bound in control cells and 144 h post BRG1 depletion (n = 3). Vinculin served as the soluble unbound control and H2A as the chromatin bound control. **e** Representative Western blot of co-IPs for BRG1, AR, FOXA1 and IgG control, alongside the supernatant (unbound fraction) and input representing 1% of the total protein in each sample (n = 3). Samples were collected in basal cell culture conditions in control cells

We next sought to further explore commonalities in the genes significantly up- or down- regulated at both time points. We used ‘Enrichr’ [85, 86] to determine which transcription factors had enriched binding at the promoters of these genes in existing ChIP-seq datasets. We discovered that the most significantly enriched datasets were for the AR and FOXA1 (Fig. 3a), both of which are important for prostate cancer growth [66, 67, 87–91]. To investigate the potential coordinated function of these transcription factors with BRG1, we compared the ChIP-seq signal of BRG1 [91], AR [87] and FOXA1 [87] at BRG1 genome-wide binding sites in LNCaP cells in basal cell culture conditions. We found the profiles separated into three clusters. Cluster 1 sites displayed strong AR and FOXA1 binding, cluster 2 had moderate AR and strong FOXA1, and cluster 3 had minimal to no signal for AR or FOXA1 (Fig. 3b). Notably we found binding of all three factors at the promoters of BRG1 regulated genes *KLK2* and *PCAT1*, and binding of BRG1 and FOXA1 upstream of the internal 3-prime promoter of *VAV3* (Fig. 3c).

To investigate the cooperative relationship between BRG1, AR and FOXA1 in regulating gene expression, we depleted BRG1 for 144 h and assessed the abundance of AR and FOXA1 recruited to chromatin using cellular fractionation followed by Western blotting. We found that while the majority of AR was unbound, the small fraction of chromatin bound AR was reduced following BRG1 knockdown (Fig. 3d). Additionally, we found that FOXA1 was considerably depleted from the chromatin after BRG1 loss. Our RNA-seq data, as well as total protein levels detected by Western blot, suggest that BRG1 does not significantly alter the overall expression of AR or FOXA1 (Fig. 3d and Additional file 2: Figure 2B-C). Together, suggesting that BRG1 is required for these transcription factors to be loaded onto the chromatin without affecting their overall abundance.

Previous research has shown that BRG1 interacts with AR and FOXA1 in hormone dependent cancers [66, 68, 92, 93]. Therefore, we preformed co-immunoprecipitations (co-IP) to see if this interaction was present in our LNCaP model. We found a modest interaction between AR and BRG1 in the AR co-IP, but no interaction between BRG1 and FOXA1, suggesting that in the basal cell culture conditions used in our model these factors do not interact sufficiently to be detected by co-IP (Fig. 3e). However, given the overlap of the ChIP-seq binding profiles and the fact that BRG1 depletion does not cause concomitant reduction in total AR and FOXA1, we hypothesise that there is either sequential binding of these factors or they are binding in close proximity without directly interacting.

### BRG1 binding is associated with the expression of DNA replication genes

**Fig. 4:**
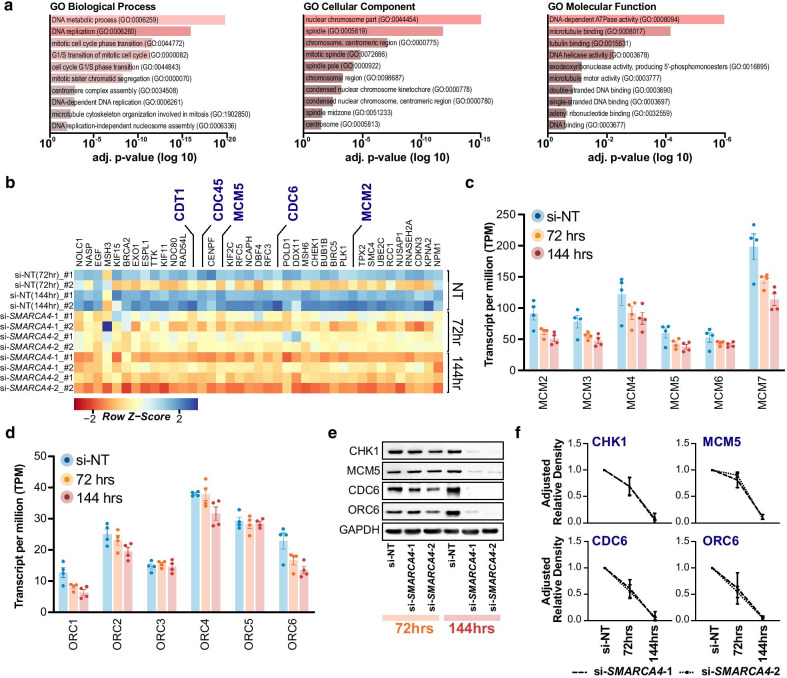
BRG1 regulates genes involved the cell cycle. **a** Gene set enrichment analysis using ‘Enrichr’ of down-regulated genes at 144 h post BRG1 knockdown. Enriched GO terms are classified as biological processes, cellular component or molecular function. Adjusted p value (log 10, reversed x-axis) of the 10 most significant GO terms is shown. **b** Heatmap of gene expression profiles from the top 40 differential cell cycle genes after BRG1 depletion. Expression is shown as the normalised row Z-score of transcripts per million reads (TPM), with blue indicating higher expression and red indicating lower expression. Genes involved in DNA replication initiation are indicated in blue. **c**, **d** Gene expression from RNA-seq (TPM), of the MCM2-7 helicase components (top) and the origin of replication complex (ORC) subunits (bottom). Bars denote mean, and error bars are SD. **e** Representative Western blot showing protein levels of replication initiation genes MCM5, CDC6 and ORC6, along with CHK1, after 72 and 144 h post BRG1 depletion. **f** Quantification of Western blots demonstrating adjusted relative density to GAPDH (n = 2). Error bars are SD

As the majority of significant gene changes occurred at 144 h post-knockdown, we next investigated the gene regulatory networks that were altered at this time point. Gene ontology analysis with Enrichr [85, 86] identified several significant (FDR < 0.05) GO terms pertaining to biological processes, cellular component and molecular function that were all broadly related to the cell cycle for down-regulated genes (Fig. 4a). However, there were no significant common processes related to the up-regulated genes. As BRG1 has previously been shown to interact with cell cycle master regulators, such as Rb and p53 [19, 94–96], we explored the relationship between the cell cycle and BRG1 further in our datasets. We compiled a list of 250 genes related to cell cycle processes, curated from the cell cycle GO terms, and of these examined the top 40 most significantly down*-*regulated genes in this list from our dataset. Of note among the list were several key genes involved in DNA replication initiation such as *CDC6*, *CDT1* and *CDC45*, as well as the Minichromosome Maintenance (MCM) replicative helicase components *MCM2* and *MCM5* (Fig. 4b). To investigate if the effect on replication initiation gene expression was more widespread, we reviewed the gene expression of the other components in the MCM2-7 replicative helicase and the origin recognition complex (ORC) and found that several of these genes were also down-regulated (Fig. 4c, d). We confirmed the down-regulation of MCM5, CDC6 and ORC6 via Western blot, along with cell cycle regulator CHK1, which revealed almost undetectable expression by 144 h post BRG1 knockdown (Fig. 4e, f).

We investigated whether AR and FOXA1 were also colocalised with BRG1 at DNA replication genes. We examined the ChIP-seq binding profiles of AR, FOXA1 and BRG1 at the promoters of 91 DNA replication genes (determined from the DNA replication GO terms) that were expressed in LNCaP cells. We found that promoters of DNA replication genes containing the active histone marks H3K4me3 and H3K27ac also displayed a weak BRG1 ChIP-seq signal, but were completely absent of AR and FOXA1 ChIP-seq signal (Additional file 3: Figure 3A), for example at the promoters of *CDC45*, *ORC6* (Additional file 3: Figure 3B). Additionally, we also note this pattern at a putative enhancer region within the *MCM2* gene (Additional file 3: Figure 3B). Our data suggest that BRG1 binding is associated with the expression of DNA replication genes in prostate cancer cells that is independent of AR and FOXA1.

### BRG1 depletion reduces proliferation

**Fig. 5:**
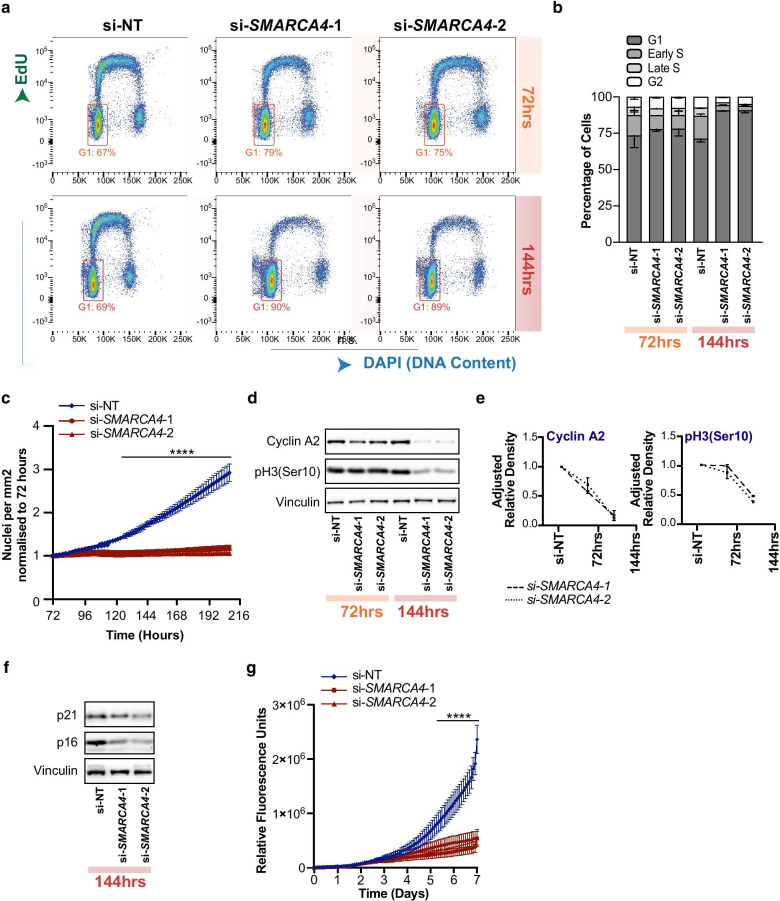
BRG1 depletion reduced proliferation. **a** Representative flow cytometry scatter of DAPI (x-axis) and EdU (y-axis) fluorescence intensity at 72 and 144 h post BRG1 knockdown. G1 cells are shown by boxed gate. **b** Percentage of cells in each phase of the cell cycle from flow cytometry data, error bars show standard deviation (n = 2). Error bars show SD. **c** Fold change of nuclei count per mm^2^ from 72 to 216 h after BRG1 knockdown, normalised to 72 h (n = 3). A significant difference was identified from 128 h until the end of the time course. Significance was determined by one-way ANOVA, with Dunnett’s multiple correction where **** = *p* < 0.0001 for si-NT versus si-*SMARCA4*-1 and si-NT versus si-*SMARCA4*-2. **d** Representative Western blot showing protein levels of mitosis markers Cyclin A2 and pH3 (Ser10) 72 and 144 h post BRG1 depletion. **e** Quantification of Western blots demonstrating adjusted relative density to Vinculin (n = 3). Error bars are SD. **f** Representative Western blot of senescence markers p21 and p16 at 144 h post BRG1 depletion (n = 3). **g** Relative fluorescence units of caspase-3 activity determined from live-cell imaging over the course of 7 days from siRNA transfection. A significant difference was detected between the si-NT cells and BRG1 depleted cells from 128 h until the end of the time course. Significance was determined by one-way ANOVA, with Dunnett’s multiple correction where **** = *p* < 0.001 for si-NT versus si-*SMARCA4*-1 and si-NT versus si-*SMARCA4*-2

Given BRG1 regulates several genes involved in proliferation and replication; we next asked if BRG1 depletion would alter cell cycle progression in LNCaP cells. We investigated this utilising the same siRNA-mediated approach to target BRG1 by depleting *SMARCA4* and conducted flow cytometry cell cycle analysis at 72 and 144 h post-knockdown. We detected an increase of cells in G1 at 72 h, which was enhanced by 144 h. Specifically, at 144 h post BRG1 depletion there was ~ 20% increase of cells in G1 and equivalent loss of cells in S phase (Fig. 5a, b). To confirm the increase in G1 population was indicative of a reduction in proliferation, we tracked the growth of LNCaP cells stably transformed with H2B-tagged mCherry using live cell imaging. We counted nuclei based on H2B–mCherry fluorescence and found that the controls cells continued to proliferate across the entire time course which we extended to 216 h from the point of siRNA transfection (Fig. 5c). However, the growth of the BRG1 depleted cells began to slow from ~ 48 h post-knockdown and showed no increase in the number of nuclei from 72 h until the end of the time course (Fig. 5c). Moreover, Western blot of mitosis markers Cyclin A2 and phosphorylation of Serine10 on H3 was significantly reduced at 144 h post BRG1 depletion (Fig. 5d, e).

We investigated putative mechanisms underlying reduced proliferation in BRG1 depleted cells. Western blots of senescence regulators p21 and p16 showed no increase at 144 h post BRG1 knockdown, indicating that senescence was not activated (Fig. 5f). We also monitored the activity of caspase 3 in live fluorescence imaging as an apoptosis readout. LNCaP cells were monitored for 24 h before treatment with a combination of 50 µg/ml of tumour necrosis factor-α (TNFα) and 100 nM of SM164 to induce apoptosis, or 2 µM of the DNA polymerase inhibitor aphidicolin to induce S-phase arrest. As expected, TNFα and SM164 induced apoptosis within 24 h (Additional file 4: Figure 4A). Apoptosis was also observed in untreated or control siRNA transfected cells starting at four days in culture (Fig. 5g, Additional file 4: Figure 4A). However, aphidicolin treatment or BRG1 depletion suppressed the delayed apoptosis (Fig. 5g, Additional file 4: Figure 4A). Our RNA-seq data demonstrate that BRG1 depleted cells maintain caspase 3, caspase 8 and caspase 9 expression (Additional file 4: Figure 4B), indicative of an intact apoptosis pathway. The most likely interpretation of these data is that culture crowding in untreated or control siRNA cells becomes lethal, and that slowing proliferation with BRG1 depletion or aphidicolin rescues apoptosis induction. Therefore, neither apoptosis nor senescence was responsible for reduced proliferation in BRG1 depleted cells. We anticipate the observed reduction in proliferation stems from a failure to initiate replication, consistent with down-regulated CDC6, ORC5 and MCM proteins.

## Discussion

Here, we examined the involvement of the SWI/SNF chromatin remodeller BRG1 and its associated encoding gene *SMARCA4* in prostate cancer transcriptional deregulation. We found that over expression of *SMARCA4* commonly occurs in both the TCGA prostate cancer cohort, irrespective of tumour subtype, and in a panel of prostate cancer cell lines. We also found that knockdown of the *SMARCA4* gene, and consequently the BRG1 protein, results in down-regulation of pro-proliferative transcriptional pathways. These included genes already known to promote prostate cancer proliferation, as well as cell cycle and DNA replication genes. Reduction of gene expression in these pathways was concomitant with G1 arrest. Taken together, our results provide new insights into BRG1′s contribution to transcriptional patterns relating to proliferation in prostate cancer.

We have demonstrated that *SMARCA4* mRNA over expression is a universal feature of prostate cancer. Clinical datasets have shown that BRG1 protein levels are over-expressed in prostate cancer, in the absence of consistent significant deleterious genetic mutations evident in *SMARCA4* [42, 44–46]. Using the large prostate cancer cohort from TCGA [50], we found that *SMARCA4* was significantly over-expressed. Consistent with this, *SMARCA4* expression was increased in a panel of both prostate cancer and transformed cell lines. These data emphasise that the overall increased expression of *SMARCA4* is a characteristic of prostate cancer, irrespective of subtype.

BRG1 depletion followed by RNA-seq revealed multiple transcriptomic alterations that were regulated by BRG1 and related to proliferation. BRG1 depletion primarily resulted in the down-regulation of BRG1′s target genes, indicating that the main role of BRG1 is to promote active gene expression. Within the down-regulated genes were genes associated with increased proliferation in prostate cancer including *KLK2*, *PCAT-1* and *VAV3*. KLK2 is a known activator of PSA, which is an important biomarker of prostate cancer, and associated with decreased apoptosis [77, 84]. *PCAT-1* promotes proliferation through the oncoprotein Myc [69, 81], while VAV3 regulates AR activity to stimulate growth in prostate cancer [78–80, 82]. Both *PCAT-1* and VAV3 are correlated with disease progression. Through an analysis of gene ontologies, we also found several cell cycle gene pathways were down-regulated with BRG1 depletion. This included numerous genes involved in DNA replication, which were among the most significantly down-regulated genes following BRG1 depletion. BRG1 is known to have a role in driving self-renewal and malignancy in B-cell acute lymphoblastic and acute myeloid leukaemias, cancers which also have over expressed BRG1 [22, 24]. Specifically, these leukaemias require high levels of BRG1 for de-condensation of the cell-specific *MYC* enhancer. In these cancers, a loss of BRG1 causes a reduction of enhancer–promoter interactions, reduced transcription factor occupancy and DNA looping which in turn reduces *MYC* expression [24]. This implies that the overexpression of BRG1 contributes to driving oncogenic transcriptional programmes which influence the proliferation capacity of cancer cells.

Our data revealed that BRG1 co-regulates the promoters of proliferation associated genes (*KLK2*, *PCAT-1* and *VAV3*) along with AR and FOXA1, and that these genes become down-regulated across our experimental time course of BRG1 depletion. Co-regulation of transcription by AR and FOXA1 in prostate cancer is associated with reprogrammed binding of AR and oncogenic patterns of gene expression that are essential for AR-driven proliferation [92, 97]. Additionally, there is a high overlap of these reprogrammed AR binding sites between LNCaP cells and primary prostate tumour tissue [97]. Here, we have shown BRG1 gene regulation overlaps with these transcription factors at gene promoters, which is concomitant with expression of prostate cancer associated genes. We also demonstrated that depletion of BRG1 affects the level of chromatin binding for both AR and FOXA1. However, we did not find significant direct interaction by co-IP. This differs from direct interactions between these proteins observed others in previous works [66, 68, 92, 93] likely due to different experimental approaches. For example, we have investigated BRG1 interactions in basal cell culture conditions, while for example, Stelloo et al. [92] looked at AR interactions in the presence of synthetic androgens.

It is noteworthy that BRG1 depletion altered the expression of DNA replication genes through a mechanism that appears independent of AR and FOXA1, suggesting that BRG1 has additional roles in other gene regulatory networks. As BRG1 is known to regulate cell cycle genes in other cancers, such as in leukaemia [24] and breast cancer [98], we speculate it is possible that regulation of cell cycle and DNA replication genes may be a general feature of BRG1 over expression in cancer, while genes co-regulated by BRG1, AR and FOXA1 are important in a prostate cancer context.

## Conclusions

In summary, our data identify fundamental role for BRG1 in maintaining active transcription for proliferation of prostate cancer cells. We find that BRG1 promotes gene expression in prostate cancer models with varying degrees of dependence on AR and FOXA1. BRG1 is required to drive the expression of numerous prostate cancer specific genes in an AR/FOXA1 dependant manner, but also works independently to drive the expression of pro-proliferative and DNA replication genes. These results provide important functional information regarding the role of BRG1 controlling proliferation in prostate cancer cells.

## Materials and methods

### Cell culture and siRNA transfection

Normal prostate epithelial cells (PrEC) (Cambrex Bio Science, CC-2555) were cultured in PrEBM (Clonetics, CC-3165) according to the manufacturer’s protocol. Briefly, PrEC cells were seeded at 2500 cells per cm^2^ and medium was replaced every two days. Cells were passaged at approximately 80% confluence. To passage a T75 flask, PrEC cells were rinsed in 6 ml Hanks balanced salt solution (Thermo Fisher Scientific, 14025076) then detached with 2 ml pre-warmed 0.025% trypsin–EDTA and incubated at room temperature for 5 min. Trypsin was inactivated with 12 ml of trypsin-neutralising solution (Clonetics, CC-5002), and cells were centrifuged at 300×*g* for 5 min. The supernatant was aspirated, and the cell pellet was re-suspended in PrEBM. The number of cells was determined on the Countess automated counter and was re-seeded at the appropriate density based on experimental needs. Cells were discarded after ~ 16 population doublings.

PC3 cells (ATCC, CRL-1435) were maintained in RPMI medium (Gibco, 11875-093) with 10% FBS, 11 ml of 1 M HEPES (Gibco, 15630080) and Pen/Strep. LNCaP cells (ATCC, CRL-1740) were cultured using custom T-Medium from Gibco (DMEM low glucose (GIBCO, 31600-034), Kaighn’s modified F-12 medium (F-12 K, 211227-014), insulin 500 × bovine pancreas (Sigma I1882), T3 6.825 ng/ml Tri-iodothyronine (Sigma-Aldrich, T5516), Transferrin 500× (Sigma-Aldrich, T5391), Biotin 500× (Sigma-Aldrich, B4639), Adenine 500× (Sigma-Aldrich, A3259)). Both prostate cell lines were cultured under recommend conditions; 37 °C with 5% CO_2_. When the cells reached ~ 80% confluence they were passaged or seeded as per experimental requirements. For siRNA transfection, LNCaP cells were seeded into 6-well plates at a density of 2.5 × 10^5^ cells per well or 10 cm dishes at 1.5 × 10^6^ cells per dish. The cells were transfected with either on target *SMARCA4* siRNA (Horizon, J-010431-06-0005 [si-*SMARCA4*-1] or J-010431-07-0005 [si-*SMARCA4*-2]) or the non-targeting control siRNA pool (Horizon, D-001810-10-05 [si-NT]) 24 h after seeding the cells using DharmaFECT 2 (Thermo Scientific, T-2002-03) as per the manufacturer’s instructions. To maintain the knockdown over a 6-day period, at 72 h post-transfection the cells were harvested, split at a ratio of 1:2 into two new wells and reverse-transfected with siRNA. The cells were then incubated for a further 72 h before collection.

### Viral transduction and selection of H2B–mCherry LNCaP cells

Replication incompetent lentiviral particles were produced by transfecting the pLXSN-H2B–mCherry plasmid (kindly provided by A. J. Cesare) into Phoenix cells and collecting the supernatant after 48 h. LNCaP cells were seeded into 6-well plates in antibiotic free media at 2 × 10^5^ cells per well. After 24hrs, 2 mL of viral supernatant, mixed with fresh media at 1:1 ratio, was added to the cells. This was replaced with fresh media after 24 h and the cells were allowed to recover for a further 24 h before checking for the presence of mCherry fluorescence. mCherry positive cells were selected for with 600 μg/ml of G418 (Sigma-Aldrich, A1720). Cells were monitored for cell death and media replaced and cells passaged as necessary. After 7 days, G418 was reduced to 50%; the cells were maintained in G418 for live cell imaging experiments.

### Growth assay

siRNA knockdown was performed as described for LNCaP cells containing the stable expression of mCherry–H2B fusion protein. At the point of siRNA transfection, the cells were placed in the Incucyte live cell imaging system (Sartorius). Images were taken every 2 h for 216 h with 16 images captured per well of a 6-well plate. The mean score each of the 16 photos per well was reported as the number or nuclei per mm^2^. Relative fold change was calculated by the difference between 0 h and each two-hour window up to 72 h and then after re-seeding the cells between 72 h and each two-hour window until the end pf the time course. A significant change was calculated by one-way ANOVA, with each BRG1 on-target siRNA compared to the non-targeting control. The results were corrected for multiple comparisons with Dunnett’s test. A significant change is defined as ****p < 0.0001*.*

### Caspase-3 apoptosis assay

LNCaP cells were seeded in a 96-well plate at 4000 cells per well, with up to 8 technical replicates per condition. After 24hrs, all cells were treated with NucView Caspase-3 Enzyme Substrate 488 (Biotium, 10402), and appropriate wells also treated with siRNA as described above, then placed in the Incucyte live cell imaging system (Sartorius). One image per well was taken every 2 h for 7 days. After 24 h in the Incucyte, the positive control cells were treated with either 50 µg/ml of TNFα (Sigma-Aldrich, H8916) + 100 nM of SM164 (Selleckchem, S7089), or 2 µM of Aphidicolin (Sigma-Aldrich, A0781). Integrated fluorescence intensity was calculated within the Incucyte Zoom software using the summed pixel intensity in calibrated units (CU) to determine the relative fluorescence units per image with the follow equation; CU x µm^2^/image. A significant change was calculated by one-way ANOVA, with each BRG1 on-target siRNA compared to the non-targeting control. The results were corrected for multiple comparisons with Dunnett’s test. A significant change is defined as ****p < 0.0001*.*

### Quantitative real-time PCR (qRT-PCR)

RNA was extracted with TRIzol reagent (Thermo Scientific, 15596026), according to the manufacturer’s protocol. Extracted RNA was re-suspended in 30 μl of nuclease-free water and quantified on the NanoDrop spectrophotometer (Thermo Scientific). cDNA synthesis was carried out with 500 ng of RNA using the SensiFAST cDNA Synthesis Kit (Bioline, BIO-65054) according to the manufacturer’s instructions.

qRT-PCR was carried out on the CFX384 Touch Real-Time PCR Detection System (Bio-Rad). A master mix was made for each qRT-PCR target containing 5 μl of KAPA Universal SYBR Fast PCR mix (KAPA Biosystems, KK4602), 0.6 μl of 5 μM forward primer, 0.6 μl of 5 μM reverse primer and 1.8 μl of nuclease-free water per reaction. Reactions conditions were 95 °C for 3 min, followed by 45 × cycles of 95 °C for 3 s and 60 °C for 30 s, then a melt curve analysis (65–95 °C, increasing at a rate of 0.5 °C every 5 s). Primers to detect *SMARCA4* were CAGAACGCACAGACCTTCAA (forward) and TCACTCTCCTCGCCTTCACT (reverse) and for detection of *18S* GGGACTTAATCAACGCAAGC (forward) and GCAATTATTCCCCATGAACG (reverse). Relative gene expression was calculated using ddCt and normalised to *18S*. A significant change in gene expression of *SMARCA4* between PrEC, LNCaP and PC3 cells was determined by one-way ANOVA and corrected with Tukey’s test for multiple comparisons.

### Western blot

Whole-cell lysates were collected with lysis buffer (50 mM HEPES, 150 mM NaCl, 10% Glycerol, 1% Triton-X-100, 1.5 mM MgCl_2_, 1 mM EGTA, 10 mM Pyrophosphate, 100 mM NaF, Roche protease inhibitor cocktail 1×), and protein level quantified using the Pierce BCA Assay Kit (Thermo Scientific, 23227) according to the manufacturer’s instructions. Sample reducing agent (Thermo Scientific, NP0004), loading buffer (Thermo Scientific, NP0007) and 10 μg protein were combined with water to a final volume of 25 μl. Protein samples were heated at 90 °C for 5 min then allowed to cool to room temperature. Protein samples were loaded on a NuPage Novex Bis–Tris 4–12% gel (Thermo Scientific, NP0321BOX) and electrophoresed at 100 V for 1.5 h in a 1 × MOPS buffer (50 mM MOPS (Biochemicals Astral Scientific, BIOMB03600, 50 mM Tris base, 0.1% SDS, 1 mM EDTA [pH 7.7]). Proteins were transferred to a polyvinylidene fluoride membrane (Bio-Rad, 1620177) at 30 V for 1 h using 1 × transfer buffer (25 mM Tris base, 192 mM Glycine [pH 8.3]) with 10% methanol (Sigma-Aldrich, 322415). Membranes were blocked for 1 h with 5% skim milk in TBS-T (20 mM Tris, 150 mM NaCl, 0.1% Tween 20 [pH 7.6]) at 4 °C. Primary antibodies used were BRG1 (Santa Cruz, sc-10768X), GAPDH (Ambion, AM4300), CHK1 (CST, 2360S), ORC6 (CST, 4737S), CDC6 (CST, 3387S), MCM5 (abcam, ab17967), AR (CST, D6F11), FOXA1 (CST, E7E8W), Vinculin (Sigma-Aldrich, V9131), H2A (CST, 12349S), p21 (CST, 2947S), p16 (CST, 92803S), Cyclin A2 (Abcam, ab181591) and pH3(Ser10) (CAT, 3377S). Primary antibodies were incubated on samples overnight at 4 °C with rotation. The membrane was then washed three times for 10mins each in TBS-T with rotation. Secondary antibodies goat anti-mouse (Santa Cruz, sc-2005) and goat anti-rabbit (Santa Cruz, sc-2004) were diluted in TBS-T containing 5% skim milk and incubated at 4 °C with rotation for 1 h. The membrane was washed three times for 10 min in TBS-T. The membrane was then covered with ECL solution (Perkin Elmer, NEL104001EA), incubated for 1 min at room temperature, and visualised by X-ray film or digitally imaged on the Bio-Rad ChemiDoc MP Imaging System (Bio-Rad). Adjusted relative density calculations were processed through ImageJ [99, 100].

### Chromatin fractionation

LNCaP cells were treated with siRNA as described above for 144 h. Cells were collected and resuspended in CSK buffer (10 mM Hepes–KOH [pH7.4], 100 mM NaCl, 3 mM MgCl^2^ and 0.5% Triton-X-100, freshly supplemented with 1 mM DTT and 1 × protease inhibitor cocktail). Cell suspensions were incubated for 5 min on ice and then centrifuged at 1500×*g* for 4 min at 4 °C. Supernatants containing the cytoplasmic and nuclear unbound soluble proteins were transferred to a fresh tube and frozen at − 80 °C. The pellet containing the chromatin bound proteins was washed in CSK buffer minus Triton-X-100. The pellet was then resuspended in CSK buffer. The pellet was then resuspended in CSK buffer with 0.1% triton-X-100 and chromatin bound proteins release through sonication with two rounds of 10 cycles (30 s on, 30 s off) in the Biorupter. Fractions were validated with Western blot of Vinculin (soluble/unbound) and H2A (chromatin bound).

### Co-immunoprecipitation (co-IP)

LNCaP cells were lysed with 0.5 ml of non-denaturing lysis buffer (20 mM Tris–HCl [pH 8.0], 137 mM NaCl, 1% IGE-PAL, 2 mM EDTA, 1:100 Benzonase), plus 1:10 phosSTOP/PIC on ice for 10 min. The cell suspension was mechanically dissociated by drawing it through a 27 G syringe to break apart the nuclei. Cell lysates were then centrifuged at 13,000×*g* for 15 min at 4 °C to pellet debris. The supernatant was transferred to a new tube and quantified with the BCA assay (Thermo Fisher, 23225). Protein A/G beads (Thermo Fisher, 88802) were washed three times in lysis buffer; then, protein lysates were then pre-cleared with 20 μl of the washed beads for 30 min at 4 °C. Protein lysates were then transferred to a new tube, and 0.5 g of appropriate antibody was added to each lysate (BRG1, Santa Cruz, sc-10768X; AR, CST, D6F11; FOXA1 CST, E7E8W, Mouse IgG isotype control, Thermo Fisher, 31903), and incubated for 2 h at 4 °C. Following this, 50 μl of prewashed A/G beads was combined with each sample and incubated for a further 30 min at 4 °C with shaking. The protein–antibody–bead complexes were then washed three times in cold non-denaturing lysis buffer. Proteins were then eluted in 40 μl of 2 × LDS buffer and frozen at − 20 °C. Detection of proteins that immunoprecipitated with the target was performed by Western blot, which was run with the input which represents 1% of the total protein in the co-IP.

### Flow cytometric cell cycle analysis

LNCaP cells were seeded at 1.5 × 10^6^ cells per 10 cm dish and transfected with siRNA as described. At 72 and 144 h post-transfection, the cells were treated with 10 μM EdU (Sigma-Aldrich, 900584) for 30 min. Remaining EdU was washed off the cells with PBS before harvesting cells, and then, 1 × 10^6^ cells were fixed in 70% ethanol and frozen at − 20 °C. Cells were then diluted 1 in 4 with PBS then pelleted and re-suspended in 1 ml of PBS containing 1% BSA (Sigma-Aldrich, A2058). Cells were again pelleted, re-suspended in 500 μl of click reaction mix (10 μM carboxyfluorescein TEG-azide, 10 mM Sodium L-ascorbate and 2 mM Copper-II-sulphate diluted in PBS), and incubated in the dark at room temperature for 30 min. Samples were then diluted with 5 ml of PBS containing 1% BSA and 0.1% Tween-20. Cells were again pelleted, washed with PBS and then resuspended in 500 μl of PBS containing 1% BSA, 0.1 mg/ml of RNase and 1 μg/ml of DAPI. Samples were analysed on the Canto II (BD Biosciences). Forward and side scatter were used to select a population of cells free of cell debris and doublets. Cells were analysed using B450 (FTIC – EdU positive) and B510 (DAPI) lasers. 50,000 single-cell events were recorded for each ample. FlowJo software v10.5 was used to analyse the data. Data were collected in biological triplicate.

### RNA-seq experiments

Total RNA was extracted with TRIzol reagent (Thermo Fisher, 15596026), quantified on the Qubit and quality assessed with the Bioanalyzer. An aliquot of 500 ng of total RNA was spiked with external controls ERCC RNA spike-in Mix (Thermo Scientific, 4456740) and libraries constructed with the TruSeq Stranded mRNA sample preparation kit (Illumina, 20020594) according to the manufacturer’s protocol. mRNA Libraries were quantified on Qubit and then stored at − 20 °C. Library quality and fragment size of RNA-seq libraries were assessed on the Bioanalyzer, and then, KAPA Library Quantification (KAPA Biosystems, KK4824) was performed according to the manufacturer’s protocol. The KAPA quantification results were used to dilute the libraries to 2 nM for sequencing. RNA-seq samples were sequenced for 100 cycles of paired-end reads on the Illumina HiSeq 2500 platform, with four samples multiplexed per lane of the high output run.

### RNA-seq data analysis

RNA-seq data were processed as described in Taberlay & Achinger-Kawecka et al*.* [9). Briefly, read counts were normalised with ERCC spike in controls, mapped to hg19/GRCh37 using STAR and counted into genes using the featureCounts [101] programme. GENCODE v19 was used as a reference transcriptome to determine the transcript per million read (TPM) value. Fold change was calculated within each time point as the log2 ratio of normalised reads per gene using the *edgeR* package in R. Genes with a fold change of ± 1.5 and FDR < 0.01 were considered significantly different. Volcano plots of differential expression were created in R with *ggplots2* and heatmaps with the *heatmap2* package with normalised row Z-score. PCA was performed in R using the *edgeR* package with log counts per million (logCPMS) over GENCODE v19 annotated gene coordinates and normalising the read counts to library size. RNA-seq multivariate analysis of transcript splicing (MATS) to calculate exon skipping and intron retention was performed with the MATS python package v4.0.2 [73–75]. Transcription factor and GO term enrichment was obtained from Enrichr (http://amp.pharm.mssm.edu/Enrichr/) online gene list analysis tool [85, 86].

### TCGA and prostate cell line expression analysis

Pre-processed RNA-seq data from the TCGA prostate adenocarcinoma cohort were downloaded (cancergenome.nih.gov) for both normal and tumour samples. The average of tumour (n = 486) and normal (n = 52) samples was calculated to determine mean expression. Separation of tumours by Gleason score and molecular subtype was performed in R using the associated clinical data to subset the appropriate groups. Significance was calculated for tumour versus normal using an unpaired t test. For comparison between Gleason score or molecular subtype, significance was calculated using one-way ANOVA with Dunnett’s multiple comparison correction.

Expression data for prostate cell lines from Presner et al. [69] were downloaded from http://www.betastasis.com/prostate_cancer/. Significance between normal, cancer and transformed cell lines was calculated using one-way ANOVA with Dunnett’s multiple comparison correction.

### ChIP-seq data

The following LNCaP ChIP-seq data were obtained from GEO (ncbi.nlm.nih.gov/geo/); BRG1 accession GSE72690 [91], H3K4me3 and H3K27me3 accession GSE38685 [102], H3K27ac and H3K4me1 accession GSE73785 [9]. These data were processed through NGSane pipeline as previously described [9, 102]. Pre-processed bigwig files for FOXA1 and AR were obtained from GEO accession GSE114274 [87]. Genome browser images of ChIP-seq data were taken from IGV. Heatmaps of ChIP-seq signal were created with *deeptools* [103].

## Supplementary Information

**Additional file 1**. **Figure 1**. **a** SWI/SNF subunit gene expression (TPM) from RNA-seq data. All subunits, except *SMARCA4* (shown in Fig. 2a), are not significantly altered. Bars denote mean, and error bars are SD. **b** PCA plot characterising the trend in expression profiles between the non-targeting control and after BRG1 knockdown. Each point on the plot represents an RNA-seq sample. Samples are separated by principal components 1 and 2, which together explain 58.37 % of the variance between the samples. **c** Number of skipped exons at 72 hours and 144 hours after BRG1 knockdown with si-*SMARCA4*-1 (black) and si-*SMARCA4*-2 (grey). **d** Number of retained introns at 72 hours and 144 hours post BRG1 depletion with si-*SMARCA4*-1 (black) and si-*SMARCA4*-2 (grey). **e** Sashimi plot of exons one and two of the *KLK3* gene in the non-targeting and 144 hour knockdown RNA-seq data. Arcs represent the number of split reads across the exons. Lower numbers represent increased retention of the first intron after BRG1 knockdown.

**Additional file 2**. **Figure 2**. **a** Expression of *KLK2, PCAT-1 and VAV3* in prostate cell lines grouped as normal, cancer or transformed. **b** *AR* and *FOXA1* gene expression from the RNA-seq datasets shown as TPM. Bars denote mean, and error bars are SD.

**Additional file 3**. **Figure 3**. **a** Heatmap of replication gene promoters, +/- 5kb from the transcription start site. **b** IGV images of the genes *CDC45*, *ORC6* and *MCM2*. Grey shaded regions contain ChIP-seq signal peaks for BRG1 and active histone modifications.

**Additional file 4**. **Figure 4**. **a** Relative fluorescence units of caspase-3 activity determined from live-cell imaging over the course of 7 days. Treatments were added 24 hours after initial imaging. **b** *CASP3*, *CASP8* and *CASP9* gene expression from the RNA-seq datasets shown as TPM. Bars denote mean, and error bars are SD.

## Data Availability

The BRG1 knockdown RNA-seq data generated for this study is available from GEO, accession number GSE150252.

## Abbreviations

AR: Androgen receptor
BRG1: Brahma-related gene 1
FOXA1: Forkhead box A1
KLK2: Kallikrein 2
MCM: Minichromosome maintenance
ORC: Origin recognition complex
PCAT1: Prostate cancer associated transcript 1
PrEC: Prostate epithelial cells
PSA: Prostate specific antigen
MATS: Multivariate analysis of transcript splicing
Rb: Retinoblastoma protein
SMARCA4: SWI/SNF-related, matrix-associated, actin-dependent regulator of chromatin, subfamily A, member 4
SWI/SNF: Switch/sucrose non-fermentable
TCGA: The cancer genome atlas
VAV3: Vav guanine nucleotide exchange factor 3
