# Supplementary figures and images for: BRG1 knockdown inhibits proliferation through multiple cellular pathways in prostate cancer
Source: Clin Epigenetics. 2021 Feb 17;13:37. doi: 10.1186/s13148-021-01023-7 (PMC7888175; doi:10.1186/s13148-021-01023-7)

SUPPLEMENTARY FIGURE 1

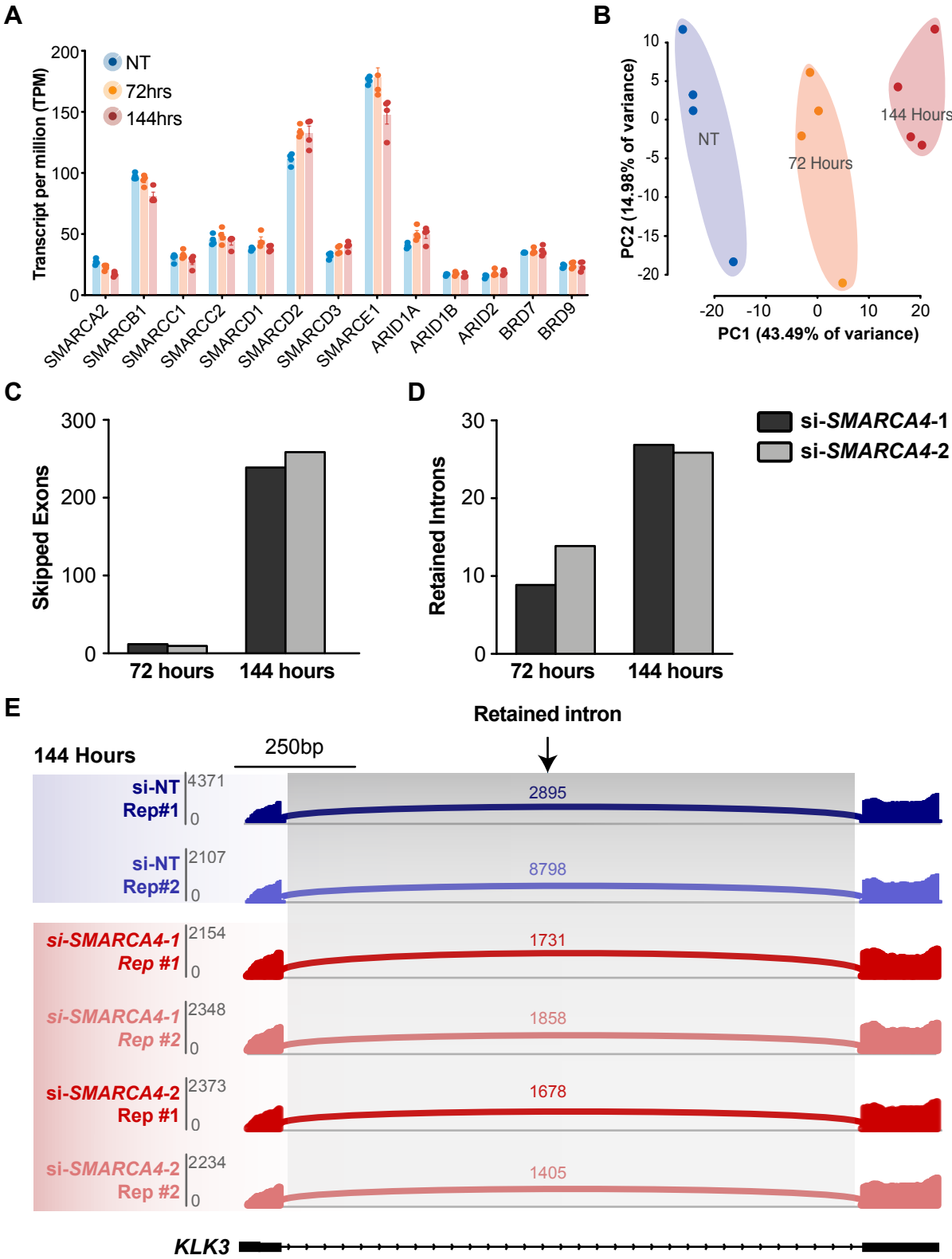

Supplement: Supplementary file 1 — Additional file 1. Figure 1. a SWI/SNF subunit gene expression (TPM) from RNA-seq data. All subunits, except SMARCA4 (shown in Fig. 2a), are not significantly altered. Bars denote mean, and error bars are SD. b PCA plot characterising the trend in expression profiles between the non-targeting control and after BRG1 knockdown. Each point on the plot represents an RNA-seq sample. Samples are separated by principal components 1 and 2, which together explain 58.37 % of the variance between the samples. c Number of skipped exons at 72 hours and 144 hours after BRG1 knockdown with si-SMARCA4-1 (black) and si-SMARCA4-2 (grey). d Number of retained introns at 72 hours and 144 hours post BRG1 depletion with si-SMARCA4-1 (black) and si-SMARCA4-2 (grey). e Sashimi plot of exons one and two of the KLK3 gene in the non-targeting and 144 hour knockdown RNA-seq data. Arcs represent the number of split reads across the exons. Lower numbers represent increased retention of the first intron after BRG1 knockdown. [file 13148_2021_1023_MOESM1_ESM.pdf]

SUPPLEMENTARY FIGURE 2

A

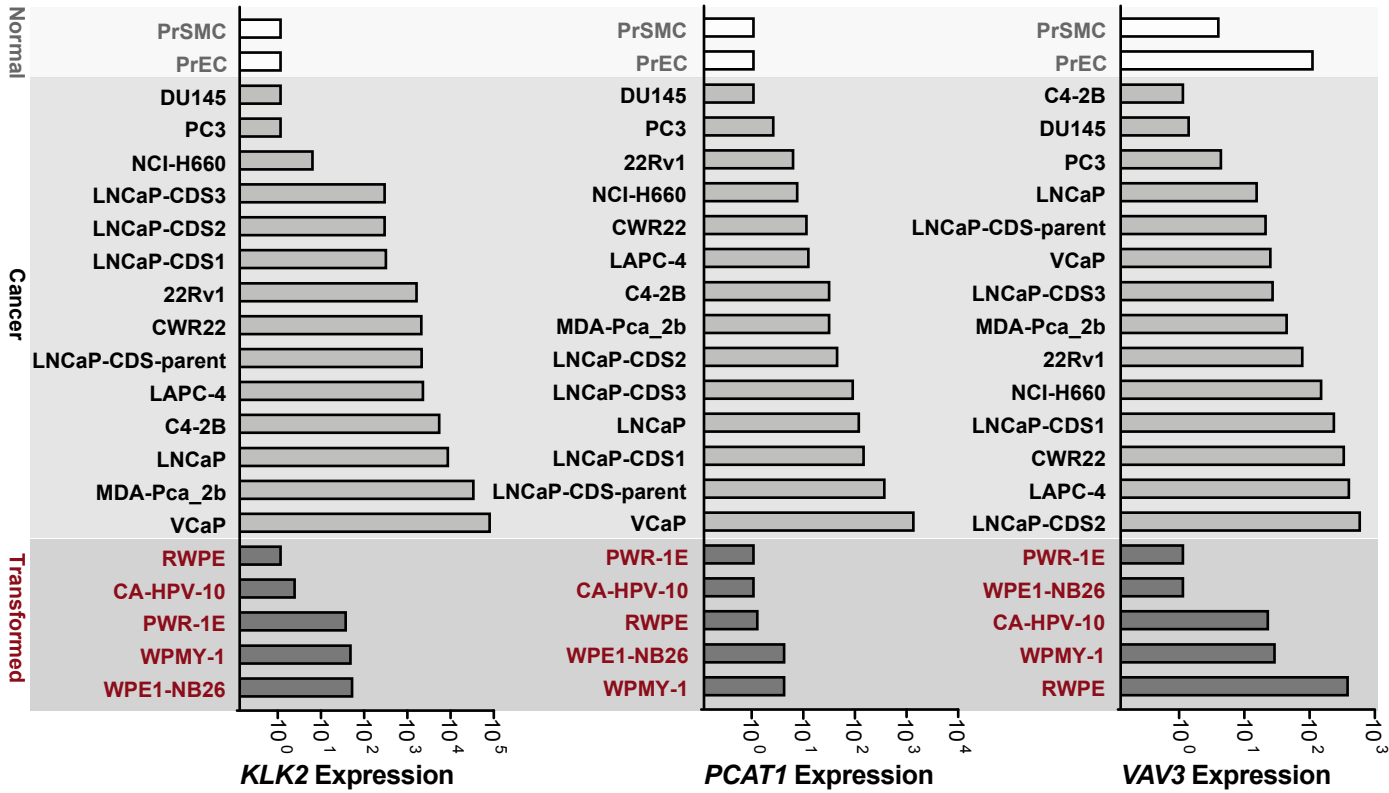

B

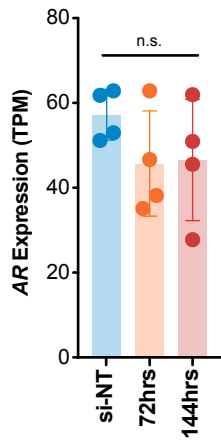

C

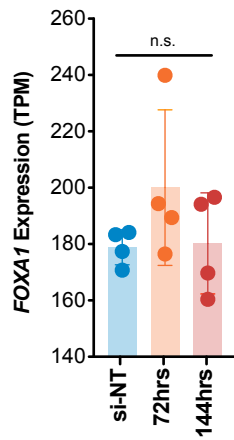

Supplement: Supplementary file 2 — Additional file 2. Figure 2. a Expression of KLK2, PCAT-1 and VAV3 in prostate cell lines grouped as normal, cancer or transformed. b AR and FOXA1 gene expression from the RNA-seq datasets shown as TPM. Bars denote mean, and error bars are SD. [file 13148_2021_1023_MOESM2_ESM.pdf]

SUPPLEMENTARY FIGURE 3

A

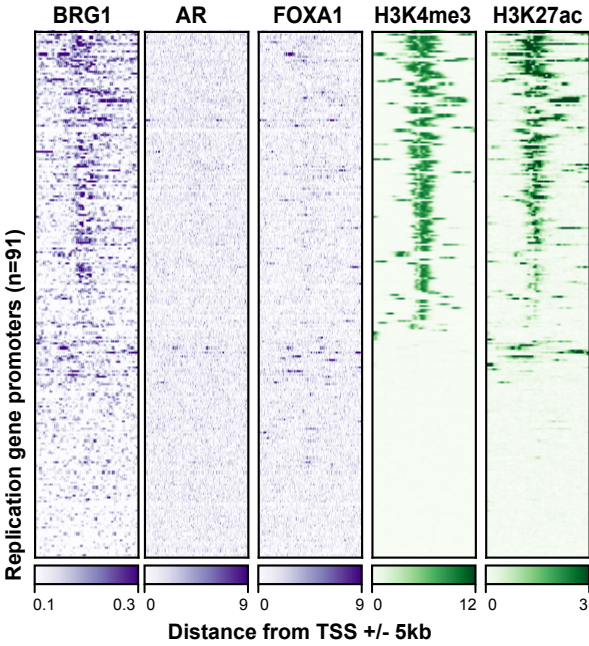

B

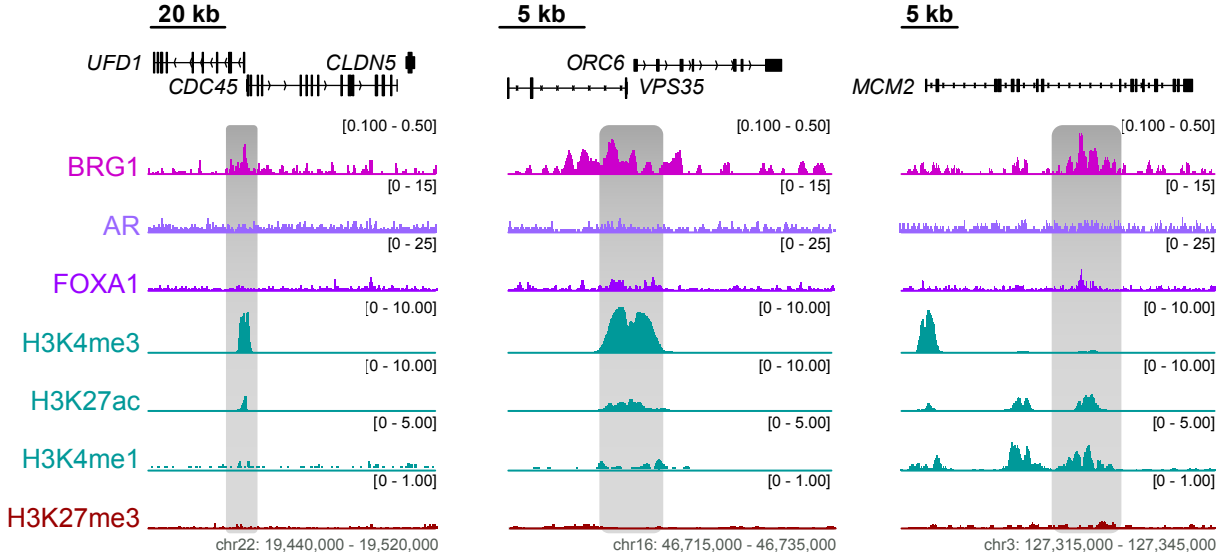

Supplement: Supplementary file 3 — Additional file 3. Figure 3. a Heatmap of replication gene promoters, +/- 5kb from the transcription start site. b IGV images of the genes CDC45, ORC6 and MCM2. Grey shaded regions contain ChIP-seq signal peaks for BRG1 and active histone modifications. [file 13148_2021_1023_MOESM3_ESM.pdf]

## SUPPLEMENTARY FIGURE 4

**A**

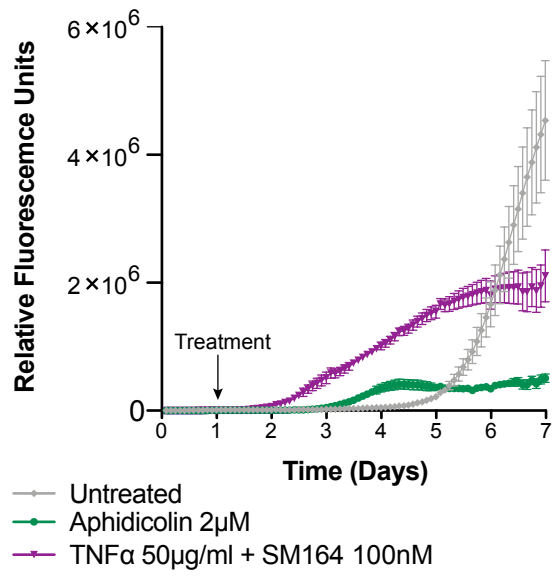

**B**

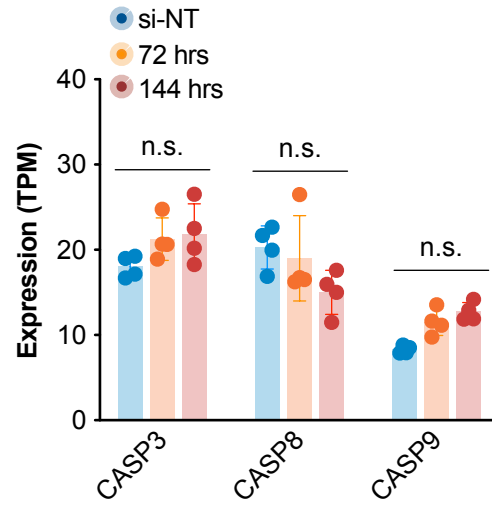

Supplement: Supplementary file 4 — Additional file 4. Figure 4. a Relative fluorescence units of caspase-3 activity determined from live-cell imaging over the course of 7 days. Treatments were added 24 hours after initial imaging. b CASP3, CASP8 and CASP9 gene expression from the RNA-seq datasets shown as TPM. Bars denote mean, and error bars are SD. [file 13148_2021_1023_MOESM4_ESM.pdf]
